# Production of Functional Fruit Snacks and Determination of Quality Characteristics

**DOI:** 10.1002/fsn3.70056

**Published:** 2025-04-18

**Authors:** Bertug Altug Arisut, Ebru Ormanli, Sebnem Tavman, Seher Kumcuoglu

**Affiliations:** ^1^ Department of Food Engineering, Graduate School of Natural and Applied Sciences Ege University İzmir Türkiye; ^2^ Department of Food Engineering, Faculty of Engineering Ege University İzmir Türkiye

**Keywords:** dietary fiber, fruit snacks, functional food, Jerusalem artichoke powder, prebiotic

## Abstract

This study aimed to explore the incorporation of Jerusalem artichoke powder (0%, 10%, 15%, and 20%) in the production of functional fruit snacks, assess the quality attributes of the resulting products, and evaluate their changes during storage. The composition, moisture content, water activity, and color values of both Jerusalem artichoke tubers and their powder were analyzed. Increasing the proportion of Jerusalem artichoke powder significantly enhanced the nutritional profile of the functional snacks, with total dietary fiber increasing from 4.68% to 9.47%, inulin content rising from less than 1% to 16.93%, protein content from 0.6% to 3.57%, and ash content from 0.83% to 3.02%. However, as the amount of Jerusalem artichoke powder and storage time increased, a reduction was observed in the total phenolic content and antioxidant activity of the samples. Slight changes in color values were noted, with decreases in L* and b* values and an increase in a* values. Moisture content ranged from 12.02% to 15.64% and decreased over the storage time, while water activity (0.51–0.65) and titratable acidity (0.95%–1.50%) remained within acceptable limits. Texture values increased with both storage time and the amount of Jerusalem artichoke powder added. Sensory evaluation indicated that the samples containing 10% and 15% Jerusalem artichoke powder were the most favored by consumers. Overall, it was concluded that Jerusalem artichoke powder can serve as a functional ingredient to enhance the nutritional value of fruit snacks. The study presents promising findings for the development of value‐added products in the dried fruit snack market.

## Graphical abstract

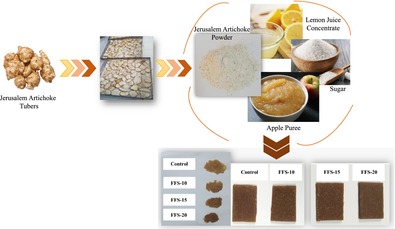

**Abbreviations**
FFS: functional fruit snacks
FFS‐10: functional fruit snacks that contain 10% JAP
FFS‐15: functional fruit snacks that contain 15% JAP
FFS‐20: functional fruit snacks that contain 20% JAP
JAP: Jerusalem artichoke powder
JAT: Jerusalem artichoke tubers
TAC: total antioxidant capacity
TPC: total phenolic content

## 1 Introduction

In the rapidly evolving world of functional foods, functional fruit snacks capture the interest of both researchers and consumers as innovative and appealing products. These snacks are distinguished by their longevity, lightweight characteristics, ease of packaging, convenience for consumption, and potential applications across various food sectors. Additionally, they boast a rich nutrient profile and interesting sensory attributes. These products are emerging as a promising way to add health benefits to daily diets, influence changes in the food industry, and increase the demand for functional foods (Sarma et al. 2023; Corfield et al. 2024; Demir and Patır 2024; Ohijeagbon et al. 2024).

In a healthy diet, the consumption of fruits and vegetables play an important role because they are rich in bioactive components such as polyphenols, flavonoids, carotenoids, and vitamins (Vázquez‐Sánchez et al. 2021; Kashyap and Sharma 2023). These bioactive components are known for their antioxidant properties and may reduce cellular damage, reduce inflammation, and have anti‐carcinogenic effects (Kashyap and Sharma 2023).

Studies have shown that functional foods derived from fruits, vegetables, and grains have health benefits. These products are preferred by various populations using renewable resources and focus on non‐waste resource‐saving technologies. The development of functional foods is an important step toward creating a sustainable and environmentally sensitive food industry, as well as contributing to improving nutrition (Perfilova et al. 2020; Sarma et al. 2023).

Fruit leather is a healthy snack that is obtained by drying the fruit puree in a layer (Vatthanakul et al. 2010; Tylewicz et al. 2020) and is rich in bioactive compounds. These fruit leather snacks may be a suitable option for individuals who want to support a healthy lifestyle and contribute to a balanced nutrition program. Research shows that such snacks can have a positive impact on overall health due to their low‐calorie content and nutritional ingredients (Demir and Patır 2024; Jethva et al. 2024).

Fruit leather production is based on pectin gelation, which is initiated or triggered by the dehydration process of fruit puree. Recent studies have tried to improve the functionality of fruit leathers by adding ingredients like folic acid, sweeteners, and protein powder. The production of leathers using various fruits such as kiwi (Barman et al. 2021), grape (Demir and Patır 2024), guava (Kurniadi et al. 2022), papaya (Addai et al. 2016), rosehip (Demarchi et al. 2018), dragon fruit (Raj and Dash 2022), mango (da Silva Simão et al. 2019), pomegranate (Tontul and Topuz 2018), banana (Sarma et al. 2023), apple (González‐Herrera et al. 2016), and apple‐plum (Nizamlioglu et al. 2022) is reported in the literature. Since apple is a good source of pectin, it represents a suitable base for developing fruit pulps (Vázquez‐Sánchez et al. 2021). Jerusalem artichoke is considered a raw material with high biological value. Jerusalem artichoke tubers stand out with their components, such as high fructose and low glucose, and high dietary fiber and pectin. These features make it a suitable alternative, especially for diabetics. Jerusalem artichoke is considered a promising raw material for the production of food additives to use in the production of sweet snacks (Zaсhesova et al. 2020; Oszmiański et al. 2021).

The aim of this study is to produce sensory‐acceptable functional fruit snacks enriched with Jerusalem artichoke powder, which is high in dietary fiber and rich in bioactive compounds, and to assess their quality characteristics throughout the storage period.

## 2 Material and Methods

### 2.1 Materials

Jerusalem artichoke tubers (JAT) were purchased from a local market in 2023, and apple puree (Brix 30%, pH 3.71, moisture content 72.92%), granulated sugar, and lemon juice concentrate (Brix 53.00%, pH 2.00, moisture content 46.04%) were obtained from Mateks Tarım Ürün. Gıda En. San. ve Tic. A.Ş. (Kemalpaşa, İzmir). Fresh Jerusalem artichoke tubers were stored at +4°C for 1 week, while apple puree and lemon juice concentrate were stored at −20°C for 1 week until use in analysis and production.

In the analyses, phenolphthalein (Norateks), methanol (Supelco, USA), sodium hydroxide (Sigma‐Aldrich, USA), Trolox (6‐hydroxy‐2,5,7,8‐tetramethyl‐chroman‐2‐carboxylic acid, Sigma‐Aldrich), sodium carbonate (Merck, Germany), Folin‐Ciocalteau reagent (Sigma‐Aldrich, USA), gallic acid (Sigma‐Aldrich, USA), potassium persulfate (Sigma‐Aldrich, USA), 2,2′‐azino‐bis(3‐ethylbenzothiazoline‐6‐sulfonic acid) diammonium salt (Sigma‐Aldrich, USA), TEMPO AC, and TEMPO YM (TEMPO bioMerieux, USA) were used.

### 2.2 Obtaining Jerusalem Artichoke Powder (JAP)

The fresh JAT were washed with a brush and sliced into approximately 3–4 mm thickness without peeling. The slices were kept in water with 1% lemon juice concentrate for 20 min to prevent enzymatic color change. Then, the drying process was performed using a pilot‐scale dryer (Eksis Makina, Isparta, Turkey) at 50°C at a speed of 1.8 m/s until it reached approximately 5% moisture content. The dried samples were ground into flour in a laboratory‐type grinder (Waring Commercial Spice Grinder, WSG60E, CT, USA) and sieved with a pore diameter of 710 μm. The resulting product was named Jerusalem artichoke powder (JAP) and was used in the production of functional snacks. JAP was stored in sterile and odorless blue vacuum packaging (PA/PE, Mateks Gıda, Turkey) at −20°C until use.

### 2.3 Production of Functional Fruit Snacks (FFS)

**FIGURE 1:**
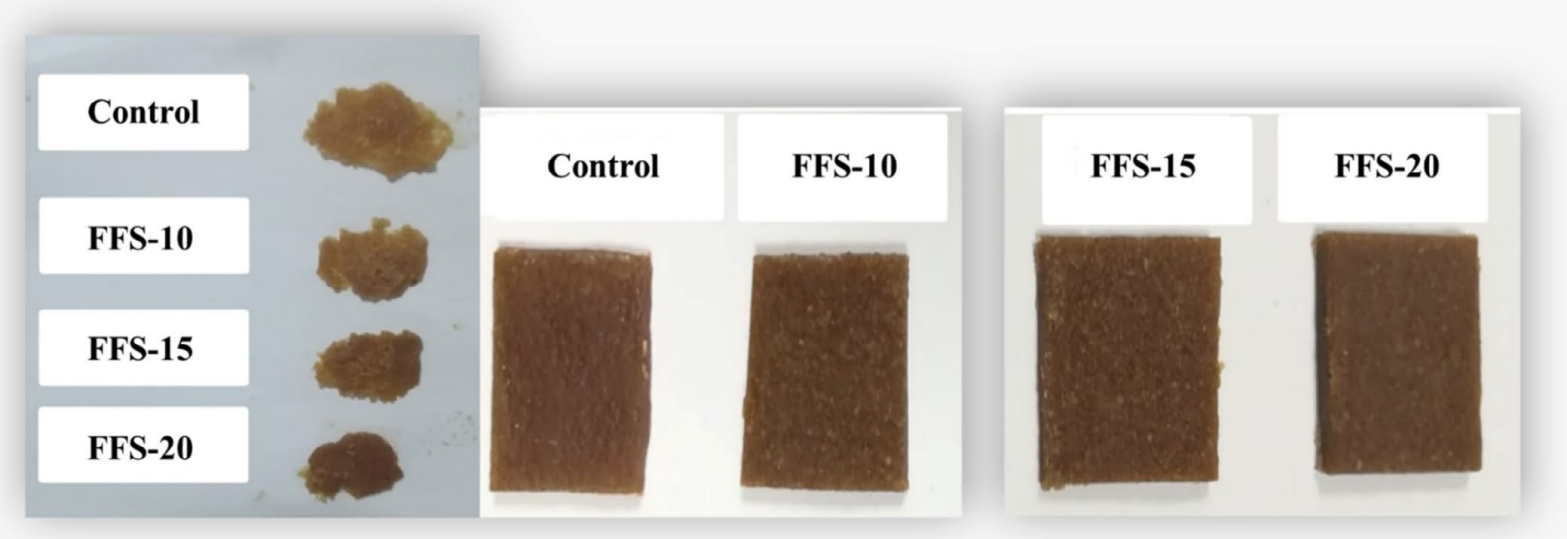
Images of snacks: control, FFS‐10, FFS‐15, and FFS‐20.

Functional fruit snacks (FFS) were produced according to the method given by Vázquez‐Sánchez et al. (2021) with some modifications such as ingredients (apple puree, sugar, JAP, and lemon juice concentrate) and a drying process which were described in detail below. During the formulation process, preliminary trials were conducted to produce 300 g of functional fruit pulp by incorporating JAP at varying concentrations ranging from 7% to 30%. As a result of these preliminary trials, the amounts of JAP to be used in the formulation were determined, taking into account both the structural properties and the amount of inulin in the final product. In this study, JAP at 4 different rates (0%, 10%, 15%, 20%) was used in the production of FFS. The formulation used in the production of fruit snacks containing JAP (for 100 g of apple puree, sugar, and lemon juice concentrate mixture) is given in Table 1. The amount of sugar and lemon juice concentrate used in all formulations was kept constant. Lemon juice concentrate was used at the rate of 0.7% of the total amount of JAP, sugar, and apple puree. The sample not containing JAP was defined as the control. After the raw materials were mixed homogeneously, the dough, each 6000 g, was poured with a thickness of 13 cm into trays and was dried in the tray dryer (Eksis Makina, Isparta, Turkey) until the moisture content of the products reached 12% ± 3% and a_w_ < 0.65. FFS were packaged using sterile and odorless blue vacuum packaging (PA/PE, Mateks Gıda, Turkey) and stored at 20°C and 55% RH for 4 months. Storage analyses were carried out once a month on the 0th, 30th, 60th, 90th, and 120th days, and the effects of storage time on the quality characteristics of the snacks were investigated. Images of FFS are given in Figure 1.

**TABLE 1:** Formulations of functional fruit snacks.

Sample code	Jerusalem artichoke powder (g)	Apple puree (g)	Sugar (g)	Lemon juice concentrate (g)
Control	0	80	20	0.7
FFS‐10	10	70	20	0.7
FFS‐15	15	65	20	0.7
FFS‐20	20	60	20	0.7

### 2.4 Analyses

#### 2.4.1 Chemical Composition

Moisture (Cemeroğlu 2010), ash (method 923.03 AOAC 2000a), protein (920.152 AOAC 1999), total dietary fiber (985.29 AOAC 2000b), inulin (HPLC‐RID method by TUBITAK) content, and water activity (978.18 AOAC 1978) analyses were carried out to determine the chemical composition of JAP and FFS.

#### 2.4.2 Titratable Acidity

Titratable acidity analyses were performed on the FFS during the storage period (4 months). About 500 mL of water was added to 5 g of the sample and mixed with a laboratory mixer (Waring Commercial Blender, 8010BU, CT, USA). The extract was obtained by passing the homogeneous mixture through coarse filter paper. Titratable acidity was determined by titrating the samples with 0.1 N NaOH until the pH value was 8.1 (AOAC 2000a, 2000b). Titratable acidity values were calculated as “g/100 mL” in malic acid equivalent.

#### 2.4.3 Color

The color values of JAP and the FFS during the storage period were determined by the method specified by Quintero Ruiz et al. (2012) and by averaging at least 15 measurements using the Minolta Chroma Meter CR‐400 (Konica Minolta Sensing Inc., Japan). Results were evaluated according to the parameters L* (luminance), a* (+ red, − green), and b* (+ yellow, − blue). Using L*, a*, and b* values, the browning index (BI) and *ΔE* value were calculated according to Equations (1) and (2), respectively.

(1) $$\mathrm{BI}=\frac{\left(x-0.31\right)}{0.172}\times 100$$

(2) $$\mathrm{\Delta E}=\sqrt{{\left(L_{0}-L\right)}^{2}+{\left(a_{0}-a\right)}^{2}+{\left(b_{0}-b\right)}^{2}}$$

#### 2.4.4 Texture

The texture of FFS was determined using the TA.XT2i Texture Analyzer (Stable MicroSystems Ltd., Godalming, Surrey, UK) during the storage period. The method given by Huang and Hsieh (2005) was modified and used to determine the texture properties of the samples. Texture profile analysis was performed on snack samples cut in 25 mm × 25 mm dimensions using a 36‐mm diameter aluminum cylindrical probe and a 50 kg load cell. Texture analysis was performed at 3 mm/s pre‐test speed, 0.5 mm/s test speed, and 0.5 mm/s post‐test speed. Samples were compressed twice to 30% of the original height, and the duration time was 5 s. Hardness, internal stickiness, elasticity, and chewiness values were calculated from the obtained two‐stage compression graph. Calculations were made by taking the average of at least 15 measurements.

#### 2.4.5 Extraction for Total Phenolic Content (TPC) and Total Antioxidant Capacity (TAC)

Firstly, the obtaining of extracts from JAP and functional snacks for use in TPC and TAC analyses was conducted with modifications to the method provided by Ertek et al. (2023). About 10 mL of 80% methanol–water solution was added to 1 g of sample. The mixture prepared with JAP was mixed for 2 min using a homogenizer (IKA, T25, Germany). The mixtures prepared with functional snacks were mixed with a magnetic stirrer for 2 h at room temperature and in the dark. After mixing, centrifugation (Hettich, Universal 200R, Germany) was applied (8000 rpm, 15 min, 4°C). The supernatant was filtered using a 0.45 μm filter and then used in the analysis of the TPC and TAC.

#### 2.4.6 Total Phenolic Content

TPC was calculated using the method given by Singleton and Rossi (1965). About 1 mL of distilled water and 1 mL of Folin–Ciocalteu reagent were added to 200 μL of extract. After the mixture was kept in the dark for 5 min, 800 μL of 7.5% Na_2_CO_3_ was added and kept for 1 h at room temperature in the dark. At the end of the period, the absorbance values were measured against a blank at 750 nm. For the calibration curve, absorbance values of gallic acid solutions were measured at different concentrations (15–300 ppm). The TPC of the samples was calculated as “mg GAE/100 g dry matter” using the equation obtained from the calibration curve (*y* = 0.0022*x* + 0.2014, *R* ^2^ = 0.9938).

#### 2.4.7 Total Antioxidant Capacity

The method described by Re et al. (1999) was applied with minor adjustments to determine the TAC values of the samples. For this purpose, ABTS (7 mM) and potassium persulfate (2.45 mM) solutions were prepared. In the preparation of the stock solution, radicalization (ABTS^•+^ formation) was carried out by mixing ABTS^•+^ and potassium persulfate solutions (1:1 v/v) and keeping it in the dark for 12–16 h. To determine the inhibition value, 2 mL ABTS^•+^ (absorbance value 0.70 ± 0.02) was mixed with a 20 μL sample and kept at room temperature and in the dark for 30 min. 80% methanol was used as a blank. At the end of the incubation, absorbance values at 734 nm were read with a Cary 60 UV–Vis spectrophotometer (Varian Inc., USA), and the TAC of samples was expressed as Trolox equivalent (μmoL TEAC/g) using the Trolox standard curve (*y* = 197.01*x* + 6.6945, *R* ^2^ = 0.9851).

#### 2.4.8 Microbial Growth

To ascertain the microbiological load change of FFS during the storage period, TEMPO AC and TEMPO YM kits (TEMPO bioMerieux, Durham, USA) were utilized for the analysis of total viable (aerobic bacteria) count and total yeast‐mold count, respectively. 10 g of the samples were weighed into a stomacher bag, and 90 mL of peptone water was added and homogenized using a stomacher. 1 mL of the resulting homogenate was taken and transferred to TEMPO AC and TEMPO YM vials containing sterile medium and sterile water (3 mL). The vials containing the samples were mixed using a vortex for 20 s, and then TEMPO AC and TEMPO YM cards were filled and incubated for 48 h at 37°C for the total viable count and 72 h at 25°C for the yeast‐mold count. At the end of the incubation periods, the cards were scanned on the device, and the microbial loads of the samples were determined. Results were given as log CFU/g sample (colony‐forming unit/g sample).

#### 2.4.9 Sensory Analyses

A hedonic test was used to determine the sensory properties of FFS during the storage period.

Sensory analysis was conducted by 15 semi‐trained panelists comprising staff, undergraduate, and graduate students from the Ege University Food Engineering Department. The samples were evaluated in terms of appearance, texture (hardness), stickiness, acidity, sourness, sweetness, and general liking using a 9‐point hedonic scale (“dislike extremely” (1) to “like extremely” (9)). Samples were coded with randomly given three‐digit numbers (Torres et al. 2015; Lawless and Heymann 2010). Ethical approval was obtained from the Ege University Scientific Research and Publication Board with protocol number 1810 for sensory analysis, and the analysis was carried out by applying all the rules that should be followed.

### 2.5 Statistical Analysis

Statistical analyses of experimental data were performed using SPSS Version 22.0 software (SPSS Inc., Chicago, IL). Whether there was a significant difference between the results was examined with a one‐way analysis of variance (One‐Way ANOVA) test within a 95% confidence interval.

Principal Component Analysis (PCA) was performed using SIMCA Software (18.0.1. Trial Version, Umetrics, Sweden) to determine the relationship between the sensory analysis results (aroma, hardness, springiness, sourness, sweetness, acidity, and general acceptability) and the results obtained by experimental methods (moisture, titratable acidity, Hardness‐T, Springiness‐T, Cohesiveness‐T, Gumminess‐T) of the functional fruit snacks. In addition, hierarchical clustering analysis (HCA) was applied to analyze potential associations between the groups. For this purpose, the distance similarity measure was determined by Euclidean distance and calculated using Ward's method.

## 3 Result and Discussion

### 3.1 Properties of Jerusalem Artichoke

Moisture, ash, protein, total dietary fiber, and inulin contents of JAT and JAP obtained from these tubers are shown in Table 2a. Water activity (a_w_) and color values (L*, a*, b*) of JAT and JAP are given in Table 2b.

**TABLE 2:** (a) Composition, (b) a_w_ and color values of JAT and JAP.

(a)					
Sample code	Moisture (%)	Protein (%)	Ash (%)	Inulin (%)	Total dietary fiber (%)
Jerusalem artichoke tubers (JAT)	81.74 ± 0.66	2.39 ± 0.08	1.04 ± 0.01	10.20 ± 0.10	1.79 ± 0.01
Jerusalem artichoke powder (JAP)	5.19 ± 0.60	10.22 ± 0.15	5.81 ± 0.18	67.6 ± 3.40	13.13 ± 0.19

(b)				
Sample code	a_w_	L*	a*	b*
Jerusalem artichoke tubers (JAT)	—	67.93 ± 6.74	3.51 ± 2.21	25.46 ± 1.57
Jerusalem artichoke powder (JAP)	0.34 ± 0.02	90.07 ± 0.59	0.20 ± 0.2	9.51 ± 0.38

### 3.2 Properties of Functional Fruit Snacks

The protein (0.60%–3.57%), ash (0.83%–3.02%), inulin (< 1%–16.93%), and total dietary fiber (4.68%–9.47%) ratios contained in the snack samples are shown in Table 3.

**TABLE 3:** Composition of functional fruit snacks.

Sample code	Protein (%)	Ash (%)	Inulin (%)	Total dietary fiber (%)
Control	0.60 ± 0.03^a^	0.83 ± 0.03^a^	< 1^a^	4.68 ± 0.11^a^
FFS‐10	2.29 ± 0.02^b^	2.37 ± 0.02^b^	10.46 ± 0.62^b^	6.85 ± 0.10^b^
FFS‐15	3.13 ± 0.03^c^	2.85 ± 0.03^c^	16.05 ± 0.10^c^	7.84 ± 0.09^c^
FFS‐20	3.57 ± 0.03^d^	3.02 = 0.01^d^	16.93 ± 0.45^c^	9.47 ± 0.12^d^

*Note:* Different letters in the same column (a, b, c, d) mean the significant differences between the groups (*p* < 0.05).

It was determined that as the amount of JAP increased in the formulation, the increase in protein and ash content was statistically significant. JAT serves as an excellent source of dietary fiber and bioactive substances because of their high content of inulin and phenolic compounds (Mu et al. 2021). Due to its prebiotic effects, inulin plays an effective role in the prevention of many metabolic disorders such as obesity, Type 2 diabetes, cardiometabolic diseases, kidney diseases, and hyperuricemia. In addition to these benefits, inulin can be used as a substitute for both sugar and fat, thus contributing to the development of alternative products (Tawfick et al. 2022). In this study, as expected, the increase in the amount of JAP used in the developed FFS leads to an increase in inulin and dietary fiber content. The amount of inulin contained in the control sample is less than 1%. The increase in total dietary fiber and inulin content is statistically significant; however, the inulin content of samples containing 15% and 20% JAP has been found to be statistically in the same group.

### 3.3 Storage Analysis

#### 3.3.1 Chemical Composition

Analyses were carried out to determine titratable acidity, a_w_, color, texture, TPC, TAC, and microbial load (mold‐yeast and total viable count) in the products during the storage period.

The moisture content values of functional snacks varied between 12.02 and 16.64 (Table 4a) and the moisture content decreased with increasing storage time statistically significant (*p* < 0.05). There are similar studies indicating that the moisture content of fruit snacks decreases with storage time (Khan et al. 2014; Ayalew and Emire 2020). The low moisture content of the product can inhibit microbial growth; thus, it can help the longer shelf‐life (Huang and Hsieh 2005; Bandaru and Bakshi 2020); however, lowering moisture contents to 10% could greatly influence the fruit leather texture (Irwandi et al. 1998). The water activity value in foods causes important chemical and biological changes to occur. High water activity values are an important criterion that affects the color, texture properties, nutritional quality, microbial growth, and stability of foods by causing undesirable reactions (Maillard, enzymatic, and other non‐enzymatic reactions, etc.). It has long been believed that one of the most crucial aspects of quality, particularly for long‐term preservation, is water activity (Fundo et al. 2015). An a_w_ value of approximately 0.61 is necessary to prevent mold and yeast growth in foods (Bhagwat 2019). It is reported in the literature that fruit leathers generally have low moisture content and medium water activity values (da Silva Simão et al. 2020). The a_w_ values obtained in this study vary between 0.51 and 0.65 (Table 4b) and are compatible with the literature.

**TABLE 4:** Moisture content (a) and the a_w_ (b) of functional fruit snacks.

Storage time (month)	Sample			
	Control	FFS‐10	FFS‐15	FFS‐20
(a) Moisture (%)				
0	15.64 ± 0.24^cB^	14.94 ± 0.03^dA^	14.62 ± 0.01^cA^	15.52 ± 0.11^cB^
1	15.54 ± 0.31^cB^	14.42 ± 0.13^cdA^	14.36 ± 0.11^bcA^	15.37 ± 0.20^cB^
2	14.86 ± 0.71^cA^	14.34 ± 0.23^cA^	14.19 ± 0.05^bA^	15.05 ± 0.00^bA^
3	13.77 ± 0.03^bA^	13.73 ± 0.03^bA^	13.98 ± 0.01^bB^	14.88 ± 0.03^bC^
4	12.02 ± 0.01^aA^	12.82 ± 0.42^aB^	12.62 ± 0.31^aAB^	13.70 ± 0.01^aC^
(b) a_w_				
0	0.61 ± 0.01^bC^	0.57 ± 0.00^aB^	0.51 ± 0.00^aA^	0.56 ± 0.01^aB^
1	0.64 ± 0.00^cB^	0.61 ± 0.00^cB^	0.56 ± 0.03^bA^	0.56 ± 0.01^aA^
2	0.65 ± 0.00^cC^	0.61 ± 0.00^cB^	0.59 ± 0.01^bcA^	0.60 ± 0.00^cAB^
3	0.64 ± 0.01^cC^	0.58 ± 0.00^bA^	0.61 ± 0.01^cB^	0.61 ± 0.01^bB^
4	0.58 ± 0.00^aA^	0.59 ± 0.00^bA^	0.58 ± 0.01^bcA^	0.59 ± 0.01^bA^

*Note:* Different letters in the same column (a, b, c, d) mean the significant differences between the months, and different letters in the same row (A, B, C) indicate the significant differences between the groups (*p* < 0.05).

Titratable acidity is an important parameter to evaluate during storage because it affects the taste, aroma, and shelf life of the product (Desrosier and Desrosier 2006). The titratable acidity values of the samples varied between 0.95% and 1.50% (Table 5). It was stated that titratable acidity increased as the amount of JAP in the samples increased (*p* < 0.05). In general, it is observed that the titratable acidity values of the samples decrease during the storage period. However, it was determined that storage time did not have a significant effect on titratable acidity (*p* > 0.05). In a study, apple, plum, and apple‐plum (50%–50%) fruit leathers were prepared, and the effects of different drying techniques (sun and oven) on their quality characteristics were determined. The titratable acidity values varied between 0.763–0.870 for apple, 1.423–1.746 for plum, and 1.303–1.328 for the mixture (Nizamlioglu et al. 2022).

**TABLE 5:** Titratable acidity of functional fruit snacks.

Storage time (month)	Sample			
	Control	FFS‐10	FFS‐15	FFS‐20
Titratable acidity (%)				
0	1.18 ± 0.07^cA^	1.25 ± 0.51^aAB^	1.41 ± 0.13^aBC^	1.49 ± 0.07^aC^
1	0.95 ± 0.13^aA^	1.22 ± 0.07^aB^	1.36 ± 0.01^aC^	1.47 ± 0.01^aD^
2	1.03 ± 0.00^abA^	1.19 ± 0.08^aB^	1.36 ± 0.05^aC^	1.48 ± 0.00^aC^
3	1.01 ± 0.00^abA^	1.20 ± 0.08^aAB^	1.34 ± 0.15^aBC^	1.49 ± 0.06^aC^
4	1.06 ± 0.05^bA^	1.14 ± 0.01^aA^	1.32 ± 0.60^aA^	1.50 ± 0.03^aA^

*Note:* Different letters in the same column (a, b, c) mean the significant differences between the months, and different letters in the same row (A, B, C, D) indicate the significant differences between the groups (*p* < 0.05).

The TAC and the TPC values of JAP were 14.26 μmol TEAC/g and 398.58 mg/100 g on a dry basis, respectively. The TAC values of the functional snacks produced within the scope of this study during the storage period are given in Table 6a, and the total phenolic content is given in Table 6b. It was determined that the TAC values of the snacks varied between 6.42 and 16.82 μmol TEAC/g, while the TPC values varied between 263.53 and 598.93 mg GAE/100 g. Although a slight decrease was observed in TAC during storage for all samples, it was determined that this change was not significant. The TPC of the control group and FFS‐10 increased with storage time, while the TPC values decreased with storage for FFS‐15 and FFS‐20. It was thought that the decrease in TAC and TPC with the addition of JAP could be due to the higher TAC and TPC values of apple puree than *those of* Jerusalem artichoke. Furthermore, the drying process used during JAP production may have contributed to a decrease in TAC and TPC.

**TABLE 6:** TAC (a) and TPC (b) of functional fruit snacks.

Storage time (month)	Sample			
	Control	FFS‐10	FFS‐15	FFS‐20
(a) Total antioxidant activity (μmol TEAC/g)				
0	16.82 ± 0.01^aD^	10.44 ± 0.05^aC^	9.24 ± 0.29^aB^	6.89 ± 0.14^aA^
1	16.69 ± 0.23^aD^	10.81 ± 0.83^aC^	8.99 ± 0.85^aB^	6.64 ± 0.32^aA^
2	16.55 ± 1.38^aC^	10.32 ± 0.41^aB^	8.70 ± 0.75^aAB^	6.66 ± 0.08^aA^
3	16.27 ± 0.65^aD^	10.16 ± 0.23^aC^	8.14 ± 0.10^aB^	6.48 ± 0.10^aA^
4	15.80 ± 0.07^aD^	9.89 ± 0.07^aC^	8.62 ± 0.10^aB^	6.42 ± 0.02^aA^
(b) Total phenolic content (mg GAE/100 g)				
0	446.58 ± 0.45^aD^	331.23 ± 2.71^aA^	426.91 ± 1.51^bC^	416.21 ± 7.12^cB^
1	466.92 ± 0.14^bC^	324.92 ± 15.12^aB^	275.97 ± 1.27^aA^	270.20 ± 0.76^abA^
2	521.67 ± 0.28^cD^	338.11 ± 3.75^aC^	280.40 ± 1.93^aB^	265.41 ± 5.46^aA^
3	593.58 ± 0.14^dC^	346.56 ± 22.10^aB^	263.53 ± 18.65^aA^	283.31 ± 0.42^bA^
4	598.93 ± 0.04^eC^	416.88 ± 0.28^bB^	278.48 ± 0.29^aA^	274.61 ± 7.39^abA^

*Note:* Different letters in the same column (a, b, c, d, e) mean the significant differences between the months, and different letters in the same row (A, B, C, D) indicate the significant differences between the groups (*p* < 0.05).

According to Buvé et al. (2018), the primary factor affecting the shelf life of fruit‐based goods is color change during storage. When the color analysis results of FFS were examined (Table 7), it was found that as the amount of JAP utilized in the formulation increased, the L* and b* values decreased and the a* value increased (*p* < 0.05). It was determined that storage time did not significantly affect the L*, a*, and b* values (*p* > 0.05), meaning that the snacks generally maintained their color stability. In a study, three cracker formulations were prepared by adding JAP at the rates of 10%, 20%, and 30%, and when the final product colors were compared, it was determined that as the proportion of JAP increased, the L* and b* values decreased, while the a* value increased (Ozgoren et al. 2019). Mphaphuli et al. (2020), mango fruit leathers were enriched with natal plum, and the properties of the leathers were determined. It was stated that the L* values of the produced leathers varied between 36.4–57.2, a* values 5.8–27.7, b* values 7.7–39.8, and ΔE values 29.6–41.3, and these results were similar to those of this study conducted.

**TABLE 7:** Color values of functional fruit snacks.

**Storage time (month)**	**Control**					**FFS‐10**				
	L*	a*	b*	**BI**		L*	a*	b*	**BI**	**ΔE**
0	35.63 ± 0.59^aC^	8.24 ± 0.12^bA^	15.42 ± 0.66^aB^	51.44 ± 1.13^aA^		34.96 ± 0.58^aC^	9.07 ± 0.04^aA^	15.00 ± 0.84^aAB^	52.65 ± 1.41^aA^	1.15 ± 0.12^aA^
1	35.62 ± 0.74^aC^	7.22 ± 0.17^aA^	15.45 ± 0.11^aA^	49.68 ± 0.13^aA^		34.91 ± 0.94^aBC^	9.18 ± 0.13^aB^	14.95 ± 1.22^aA^	52.77 ± 2.08^aA^	2.32 ± 0.36^bA^
2	35.39 ± 1.35^aC^	7.22 ± 0.39^aA^	15.56 ± 0.15^aB^	50.14 ± 0.21^aA^		34.88 ± 0.84^aBC^	9.22 ± 0.34^aB^	14.83 ± 1.10^aAB^	52.60 ± 2.30^aA^	2.35 ± 0.26^bB^
3	34.88 ± 0.80^aC^	7.21 ± 0.58^aA^	15.55 ± 0.55^aC^	50.56 ± 1.55^aA^		34.73 ± 0.74^aC^	9.23 ± 0.18^aB^	14.71 ± 1.18^aBC^	52.50 ± 2.30^aA^	2.22 ± 0.59^bA^
4	34.76 ± 0.52^aB^	7.19 ± 0.42^aA^	15.34 ± 0.62^aB^	50.16 ± 1.69^aA^		34.55 ± 0.59^aB^	9.95 ± 0.50^bC^	14.25 ± 1.39^aAB^	52.94 ± 3.54^aA^	3.04 ± 0.21^bB^
**Storage time (month)**	**FFS‐15**					**FFS‐20**				
	L*	a*	b*	**BI**	**ΔE**	L*	a*	b*	**BI**	**ΔE**
0	33.88 ± 0.13^aB^	9.17 ± 0.13^aA^	14.75 ± 0.55^aAB^	53.37 ± 1.39^aA^	1.13 ± 0.50^aA^	33.06 ± 0.53^aA^	9.30 ± 0.95^aA^	13.58 ± 0.08^aA^	51.69 ± 1.45^aA^	1.65 ± 0.26^aA^
1	33.77 ± 0.03^aAB^	9.19 ± 0.61^aB^	14.72 ± 0.97^aA^	53.45 ± 3.38^aA^	1.28 ± 0.83^aA^	32.95 ± 1.36^aA^	9.31 ± 0.35^aB^	13.51 ± 0.45^aA^	51.66 ± 0.35^aA^	1.86 ± 0.41^aA^
2	33.70 ± 0.12^aAB^	9.20 ± 0.51^aB^	14.58 ± 1.38^aAB^	53.20 ± 4.06^aA^	1.28 ± 0.59^aA^	32.93 ± 0.75^aA^	9.32 ± 0.60^aB^	13.51 ± 0.77^aA^	51.68 ± 2.22^aA^	1.50 ± 0.19^aA^
3	33.41 ± 0.53^aB^	9.27 ± 0.72^aB^	14.49 ± 0.42^aB^	53.42 ± 1.79^aA^	1.53 ± 0.33^aA^	32.42 ± 0.53^aA^	9.64 ± 1.28^aB^	13.31 ± 0.06^aA^	52.34 ± 2.04^aA^	1.65 ± 0.38^aA^
4	33.36 ± 0.78^aA^	9.66 ± 0.52^aBC^	14.20 ± 0.93^aAB^	53.51 ± 2.36^aA^	1.28 ± 0.17^aA^	33.02 ± 0.44^aA^	9.31 ± 0.15^aB^	13.06 ± 0.26^aA^	50.51 ± 0.47^aA^	1.26 ± 0.80^aA^

*Note:* Different letters in the same column (a, b, c) mean the significant differences between the months, and different letters in the same row (A, B, C) indicate the significant differences between the groups (*p* < 0.05).

The hardness values of FFS were varied between 99.31 and 301.04 N, cohesiveness values between 0.25 and –0.72, springiness values between 0.15 and 0.67, and gumminess values between 1395.39 and 6298.31 (Table 8). It was found that the texture parameters of functional snacks' hardness, cohesiveness, springiness, and gumminess were increased with the addition of JAP (*p* < 0.05). Additionally, all the texture parameters were increased with the storage time. Irwandi et al. (1998) investigated the effects of the type of packaging material on the physicochemical, microbiological, and sensory properties of durian fruit leathers dried by different methods during storage. It was found that the moisture content of both oven‐dried and cabinet‐dried durian leathers decreased significantly after the 4th week for other packaging materials except laminated aluminum foil. It has been emphasized that low moisture content may have a negative impact on the tissue quality of fruit leathers. For this reason, it was thought that the decrease in moisture content during storage may be effective in increasing the texture values.

**TABLE 8:** Texture values of functional fruit snacks.

Storage time (month)	Sample				Sample			
	Control	FFS‐10	FFS‐15	FFS‐20	Control	FFS‐10	FFS‐15	FFS‐20
	Hardness *(N)*				Springiness			
0	99.31 ± 2.66^aA^	119.03 ± 2.38^aAB^	130.53 ± 1.42^aBC^	150.09 ± 14.94^aC^	0.15 ± 0.01^aA^	0.20 ± 0.01^aB^	0.22 ± 0.01^aBC^	0.24 ± 0.02^aC^
1	110.37 ± 11.57^abA^	135.51 ± 5.07^abB^	142.97 ± 4.31^bBC^	167.06 ± 13.03^aC^	0.19 ± 0.01^bA^	0.25 ± 0.02^bB^	0.29 ± 0.02^bBC^	0.32 ± 0.01^abC^
2	126.04 ± 5.08^bA^	144.84 ± 7.76^bB^	155.86 ± 3.22^cB^	173.64 ± 3.86^aC^	0.29 ± 0.01^cA^	0.32 ± 0.02^cAB^	0.35 ± 0.01^cBC^	0.37 ± 0.01^bcC^
3	185.48 ± 14.20^cA^	207.32 ± 11.28^cAB^	216.71 ± 3.82^dB^	243.75 ± 7.68^bC^	0.34 ± 0.00^dA^	0.38 ± 0.01^dB^	0.39 ± 0.02^dB^	0.44 ± 0.01^cC^
4	211.23 ± 6.78^dA^	258.09 ± 8.54^dB^	263.10 ± 1.92^eB^	301.04 ± 5.48^cC^	0.41 ± 0.02^eA^	0.51 ± 0.01^eB^	0.53 ± 0.01^eB^	0.67 ± 0.07^dC^
	Cohesiveness				Gumminess			
0	0.25 ± 0.02^aA^	0.31 ± 0.02^aAB^	0.35 ± 0.02^aAB^	0.37 ± 0.08^aB^	1395.39 ± 114.84^aA^	2536.68 ± 49.26^aB^	2899.8 ± 257.87^aB^	3583.25 ± 192.11^aC^
1	0.31 ± 0.02^bA^	0.37 ± 0.01^bB^	0.39 ± 0.03^aB^	0.44 ± 0.00^abC^	1454.01 ± 107.5^aA^	3164.01 ± 25.53^aB^	3316.35 ± 111.46^aB^	3862.07 ± 6.78^aC^
2	0.47 ± 0.02^cA^	0.49 ± 0.02^cAB^	0.50 ± 0.01^bAB^	0.53 ± 0.02^bcB^	1956.86 ± 130.30^bA^	4088.26 ± 29.84^bB^	4428.17 ± 186.87^bC^	4759.28 ± 118.31^bC^
3	0.52 ± 0.01^dA^	0.55 ± 0.02^dAB^	0.59 ± 0.01^cBC^	0.62 ± 0.02^cdC^	2951.67 ± 65.96^cA^	4792.31 ± 504.29^cB^	5327.08 ± 23.02^cBC^	5900 ± 207.79^cC^
4	0.54 ± 0.02^dA^	0.61 ± 0.01^eAB^	0.68 ± 0.03^dBC^	0.72 ± 0.04^dC^	3108.45 ± 53.94^cA^	5239.76 ± 231.06^cB^	5891.49 ± 130.17^dC^	6298.31 ± 112.5^dD^

*Note:* Different letters in the same column (a, b, c, d, e) mean the significant differences between the months, and different letters in the same row (A, B, C, D) indicate the significant differences between the groups (*p* < 0.05).

The total mold‐yeast and total viable count results of functional snacks are given in Table 9. Mold‐yeast counts remained below 10 cfu/g for all samples during storage, and the total viable count generally increased with the increase in storage time. It is reported in the literature that fruit leathers often have microbiological stability as a result of their intermediate water activity, low moisture content, and low pH value (Torres et al. 2015). It was determined that the functional snacks produced within the scope of the study were below the log 6 value, which is the limit of suitability for consumption (Daelman et al. 2013), for all samples at the end of the 4‐month storage period. In Azeredo et al. (2006), trials were carried out to minimize the drying time required to produce mango leathers without added preservatives and sugar, and the acceptability and storage stability of these leathers were evaluated. It was stated that mango leathers with an a_w_ of 0.62 and pH values of 3.8 remain microbiologically stable for at least 6 months without the use of chemical preservatives.

**TABLE 9:** Total viable and yeast‐mold count values of functional fruit snacks.

Storage time (month)	Sample				Sample			
	Control	FFS‐10	FFS‐15	FFS‐20	Control	FFS‐10	FFS‐15	FFS‐20
	Total viable count (log CFU/g sample)				Total yeast‐mold count (log CFU/g sample)			
0	< 10	2.3×10	1.5×10^2^	3×10^3^	0	0	0	< 10
1	20	4.6×10	2.5×10^2^	3.3×10^3^	< 10	< 10	< 10	< 10
2	89	2.0×10^2^	1.8×10^3^	2.5×10^4^	< 10	10	< 10	< 10
3	38.5	3.1×10^3^	1.285×10^4^	1.45×10^4^	< 10	< 10	< 10	< 10
4	59	1.7×10^4^	4.9×10^4^	1.7×10^4^	< 10	21	21	< 10

**FIGURE 2:**
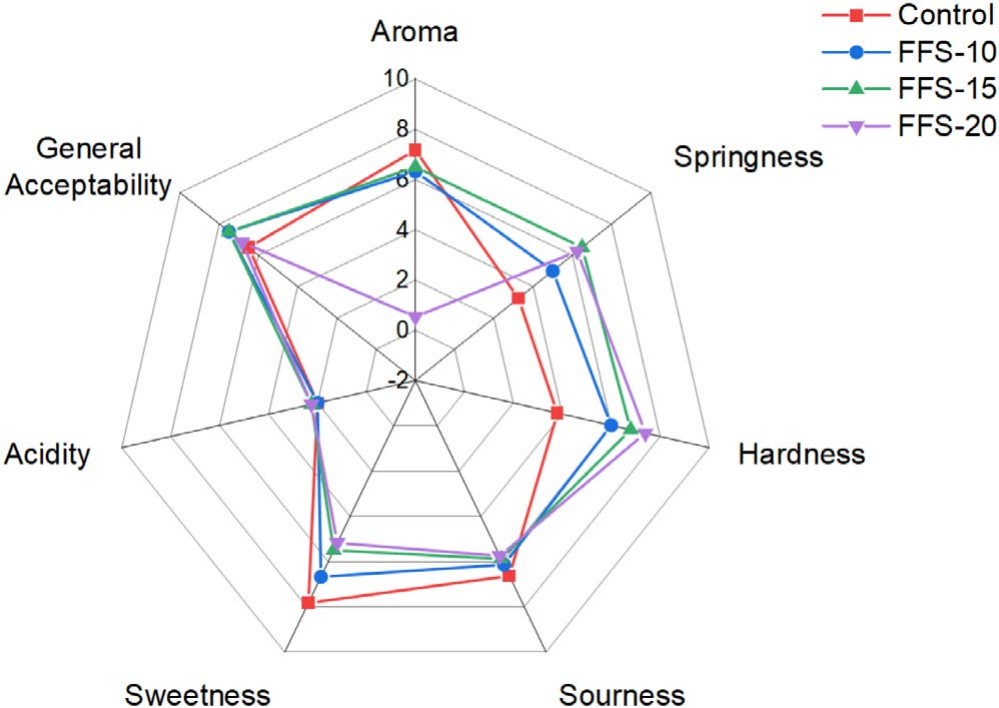
Spider graph for sensory analysis of functional fruit snacks.

It was determined that as the amount of JAP increased, consumer preferences in the aroma, sourness, and sweetness values of the samples decreased. Additionally, it was observed that the texture (springiness and hardness) and acidity values of the samples were more appreciated with the increase in JAP, and it was determined that the sensory analysis results confirmed the texture analysis results. The samples with the highest general acceptability in sensory analysis were FFS‐10 and FFS‐15, then FFS‐20 was preferred, and the control sample received the lowest score (Figure 2). Consumers have expressed a preference for products with a certain amount of JAP added. This analysis suggests that developed FFS with high dietary fiber content may be well received by consumers.

**FIGURE 3:**
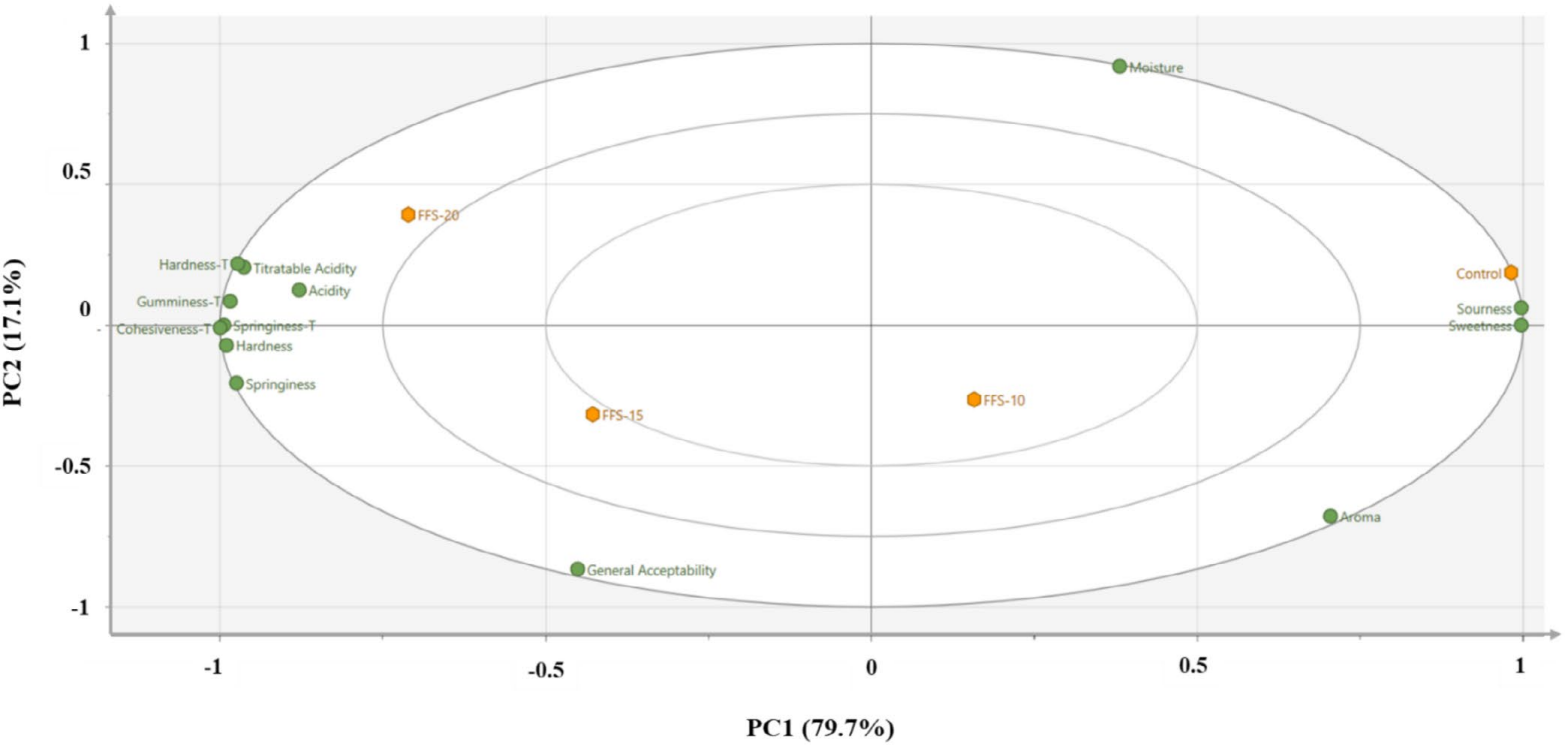
Biplot of principal component analysis (PC1) versus (PC2) of functional fruit snacks on the sensorial, physical, and chemical attributes.

PCA and HCA were used to perform a multivariate statistical evaluation of the addition of JAP on the moisture content, titratable acidity, texture analysis results (Hardness‐T, Springiness‐T, Cohesiveness‐T, and Gumminess‐T), and sensory analysis results (aroma, springiness, hardness, sourness, sweetness, acidity, and general acceptability) of functional fruit snacks. The biplot of the PCA (R2: 0.99) was given as loading and score plots (Figure 3). There are four distinct groups in the graph, and the first principal component (PC1) explains 79.7% of the overall variability in the data, while the second principal component (PC2) explains 17.1%. A clear distinction between the control and FFS samples is observed due to the influence of PC1 and PC2. The texture of the snacks evaluated through experimental and sensory analyses, as well as acidity and titratable acidity, clustered together on the same side, thus indicating a positive correlation between these variables. In contrast, sourness and sweetness are positioned on the opposite side in the loading plot, suggesting a negative correlation with all textural properties and acidity analysis results. A decreasing trend in textural properties and acidity values was noted with the addition of JAP. Furthermore, control and FFS‐20 samples exhibited a negative correlation in terms of aroma and general acceptability.

## 4 Conclusions

Fruits and vegetables are vital to a healthy diet because they are abundant in bioactive substances such as vitamins, carotenoids, flavonoids, and polyphenols. As consumer demand for healthy snacks continues to rise, researchers and industry are increasingly focusing on developing functional foods. Fruit‐based snacks, with their chewy texture and nutrient‐dense profile, align well with consumer expectations, offering both convenience and health benefits. Among potential ingredients, Jerusalem artichoke stands out due to its high biological value. In this study, functional fruit snacks were developed using JAP derived from its tubers. The snacks were prepared with varying amounts of JAP (10%, 15%, and 20%), and their composition and storage‐related changes were analyzed by physical, chemical, microbiological, and sensory analysis. The snacks' total dietary fiber, inulin, protein, and ash content significantly improved as the proportion of JAP increased. Slight color changes were noted, with L* and b* values decreasing and a* values increasing, while the browning index (BI) remained stable. Moisture, total phenolic content, and antioxidant activity decreased over the storage period, whereas texture values increased both with JAP addition and storage time. Sensory evaluation revealed that the most preferred samples were those containing 10% and 15% JAP, followed by the 20% JAP sample. The control group was the least liked, suggesting that increasing the JAP content improves consumer acceptance up to a certain level. The snacks developed in this study are considered a promising healthy alternative for the food industry, given their high dietary fiber content and favorable consumer acceptance.

## Author Contributions

**Bertug Altug Arisut:** data curation (equal), formal analysis (equal), investigation (equal), methodology (equal), software (equal), visualization (equal), writing – original draft (equal), writing – review and editing (equal). **Ebru Ormanli:** data curation (equal), formal analysis (equal), investigation (equal), methodology (equal), software (equal), visualization (equal), writing – original draft (equal), writing – review and editing (equal). **Sebnem Tavman:** conceptualization (equal), supervision (equal), writing – review and editing (equal). **Seher Kumcuoglu:** conceptualization (equal), project administration (equal), resources (equal), supervision (equal), writing – review and editing (equal).

## Ethics Statement

The Ege University Scientific Research and Publication Board granted ethical approval for the sensory analysis with protocol number 1810, and all applicable guidelines were followed in conducting the analysis.

## Conflicts of Interest

The authors declare no conflicts of interest.

## Data Availability

Data will be made available upon request.
